# Progenitor Cells Derived from Drain Waste Product of Open-Heart Surgery in Children

**DOI:** 10.3390/jcm8071028

**Published:** 2019-07-12

**Authors:** Tak-Wah Wong, Chung-Dann Kan, Wen-Tai Chiu, Kin Lam Fok, Ye Chun Ruan, Xiaohua Jiang, Junjiang Chen, Chiu-Ching Kao, I-Yu Chen, Hui-Chun Lin, Chia-Hsuan Chou, Chou-Wen Lin, Chun-Keung Yu, Stephanie Tsao, Yi-Ping Lee, Hsiao Chang Chan, Jieh-Neng Wang

**Affiliations:** 1 Department of Dermatology, National Cheng Kung University Hospital, College of Medicine, National Cheng Kung University, Tainan 704, Taiwan; 2 Department of Biochemistry and Molecular Biology, College of Medicine, National Cheng Kung University, Tainan 701, Taiwan; 3 Center of Applied Nanomedicine, National Cheng Kung University, Tainan 701, Taiwan; 4 Department of Surgery, Institute of Cardiovascular Research Center, National Cheng Kung University Hospital, College of Medicine, National Cheng Kung University, Tainan 704, Taiwan; 5 Department of Biomedical Engineering, National Cheng Kung University, Tainan 701, Taiwan; 6 Epithelial Cell Biology Research Center, School of Biomedical Sciences, Faculty of Medicine, the Chinese University of Hong Kong, Shatin, Hong Kong; 7 Key Laboratory for Regenerative Medicine, Ministry of Education of the People’s Republic of China, Shatin, HongKong; 8 Institute of Basic Medical Sciences, College of Medicine, National Cheng Kung University, Tainan 704, Taiwan; 9 Biomedical Technology and Device Research Laboratories, Industrial Technology Research Institute, Liuo-Jia, Tainan 734, Taiwan; 10 Department of Microbiology and Immunology, Center of Infectious Disease and Signaling Research, College of Medicine, National Cheng Kung University, Tainan 701, Taiwan; 11 National Laboratory Animal Center, National Applied Research Laboratories, Taipei 11529, Taiwan; 12 Department of Pediatrics, National Cheng Kung University Hospital, College of Medicine, National Cheng Kung University, Tainan 704, Taiwan

**Keywords:** congenital heart disease, cardiac surgery, open heart, progenitor cells, regeneration, stem cells

## Abstract

Human cardiac progenitor cells isolated from the same host may have advantages over other sources of stem cells. The aim of this study is to establish a new source of human progenitor cells collected from a waste product, pericardiac effusion fluid, after open-heart surgery in children with congenital heart diseases. The fluid was collected every 24 h for 2 days after surgery in 37 children. Mononuclear cells were isolated and expanded in vitro. These pericardial effusion-derived progenitor cells (PEPCs) exhibiting cardiogenic lineage markers, were highly proliferative and enhanced angiogenesis in vitro. Three weeks after stem cell transplantation into the ischemic heart in mice, cardiac ejection fraction was improved significantly without detectable progenitor cells. Gene expression profiles of the repaired hearts revealed activation of several known repair mechanisms including paracrine effects, cell migration, and angiogenesis. These progenitor cells may have the potential for heart regeneration.

## 1. Introduction

Despite the recent advances in molecular medicine and health care; cardiac diseases, including both adult and congenital heart diseases, are still the leading cause of morbidity and mortality throughout the world [1]. Congenital heart disease (CHD) is the most common congenital anomalies in newborns [2]. Globally, there are around 1.35 million neonates born with CHD every year over the last 15 years. The highest CHD birth prevalence is reported in Asian countries with 9.3 per 1000 live births which is one of the leading causes of perinatal and infant death from congenital malformations [3]. Approximately 25 percent of these neonates require surgery or catheter-based intervention in the first year of life [4]. The prognosis of CHD is good with 83% of patients are free from reoperation for 20 years and the overall survival rate was 86%, including early mortality [5]. However, multiple-stage operations are needed to correct the defects in cases with complex CHD. Lui et al. pointed out the misperception that CHD is cured by surgery [6]. CHD patients are at increased risk of developing myocardial ischemia or premature coronary artery disease (CAD) while growing up. The number of adult CHD patients continues to increase by 5% per year, there are now more than 1 million patients in the United States [6]. Owing to the lack of effective treatment strategy after severe cardiac injury in these patients, stem cell-based therapies may be a potential therapeutic strategy [7,8].

A variety of stem cell populations have been explored for their potential to promote cardiac repair and regeneration. The results are controversial since each type of stem cell has its own profile of advantages, limitations, and translational practicability. The most frequently studied stem cell populations in cardiac diseases include embryonic stem cells (ESC), bone marrow-derived stem cells (BMSC), tissue-specific stem cells (TSSC), and the most recently inducible pluripotent stem cells (iPS). ESCs are conceptually attractive for cardiac repair because of the potential to differentiate into different cell types in a damaged heart. Despite the recent success of ESCs engraftment and repairing injured myocardium in a primate model of myocardial infarction [9], translation into clinical use has been hindered by ESCs’ genetic instability, potential tumorigenic and immunogenic properties, and ethical considerations related to the origin of these cells [7]. BMSCs are composed of several cell populations that have the capacity to differentiate into various cell types, including hematopoietic stem cells (HSC), mesenchymal stem cells (MSC), endothelial progenitor stem cells (EPC), and others. Each cell type has been reported to improve cardiac function on top of standard therapy in cardiac diseases. Most clinical trials to date have used total bone marrow mononuclear cells, which comprise HSCs, MSCs, and monocytes [7]. Promising results from Cochrane Heart Group enrolled more than a thousand patients with heart diseases but debates continued because of a high degree of heterogeneity in the results [10,11]. It is still too early to draw conclusions on the efficacy of BMSCs [12]. TSSC, such as adipose tissue-derived stem cells (ADS) [13] and umbilical cord blood-derived stem cells (UBS) [14], appeared to be attractive in the treatment of cardiac diseases. Early promising results in clinical trials have been shown in ADS treated cardiac damages [13]. However, the tumorigenic potential of these cells cannot be totally excluded [13]. UBS is still in the early recruitment stages of the clinical trial [14].

Resident cardiac progenitor cells expanded from heart biopsy in the early clinical trial showed promising results [15]. These cells have exhibited robust cardiovascular differentiation potential. However, an invasive heart biopsy is not easily accepted by parents of CHD patients. Human iPS cells provide new revenue for sufficient stem cells in the treatment of heart failure. Yet the safety in clinical applications remains to be determined [16]. Taken together, while multiple clinical trials of stem cell therapy worldwide on adult or congenital heart diseases suggest that stem cell therapy is safe with modest improvement in cardiac functioning and structural remodeling [7,16], the right source of stem cells remains one of the major challenges in this field. A new source of stem cells, which is free of ethical conflict, easy to isolate and propagate, and with cardiac regenerative potential, is desirable for cell-based therapy in the treatment of CHD.

Draining of blood and effusion from the mediastinum and pleural space following open-heart surgery is critical to establish adequate evacuation of fluid after surgery [17]. Surgical drainage is usually left in place for 3 to 4 days until the fluid is decreased to less than 50 ml per day with stable hemodynamic and respiratory functions. In this study, we explored the possibility of the isolation and expansion of progenitor cells from pericardial effusion drained after open-heart surgery in children with congenital heart diseases. We named these cells pericardial effusion-derived progenitor cells (PEPCs).

## 2. Materials and Methods

### 2.1. Isolation and Expansion of Progenitor Cells from Open-Heart Surgery Drain Fluids

**Figure 1:**
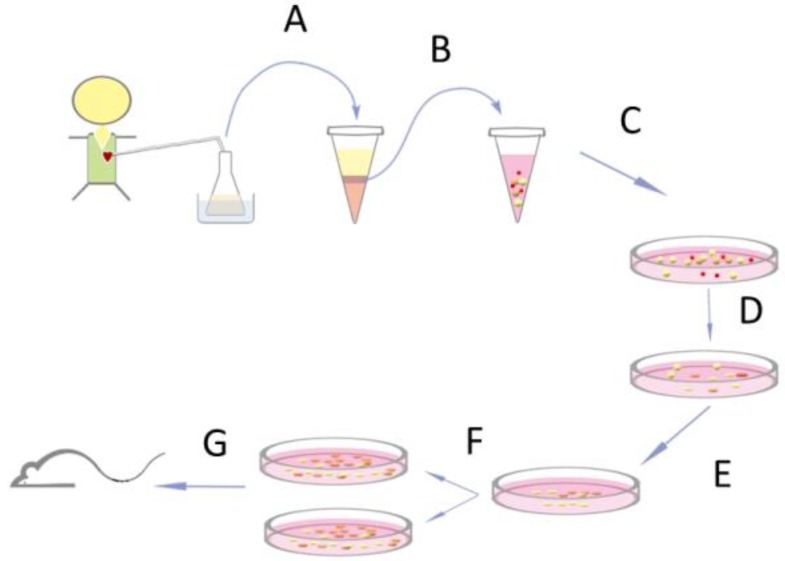
Schematic drawing of the isolation and expansion procedures for human pericardial effusion-derived progenitor cells (PEPCs). (**A**) Drain fluid was collected on the first and second day after open-heart surgery in a drain bottle containing 300 mL of 4 °C normal saline. The bottle was kept at 4 °C during collection. (**B**) Mononuclear cells were collected by density gradient centrifugation with Ficoll-Paque. (**C**) The mononuclear cells with red blood and white blood cells were plated on a fibronectin-coated plate and cultured in cardiosphere medium. (**D**) The red blood and white blood cells were removed by serial medium supplement every 3 days. (**E**) Spindle-like cells were noted as early as 3 days while cardiospheres appear around 7 days after seeding. (**F**) Further cell expansions to obtain sufficient cell number for transplantation. (**G**) PEPCs were injected into peri-infarction regions of a mouse heart.

All human studies were conducted according to the principles expressed in the Helsinki Declaration and approved by the institutional review board. Thirty-seven patients with congenital heart diseases aged 3 days to 43 months (mean 9.7 ± 9.2 months, Table S1) and scheduled for open-heart surgery were recruited after signing informed consent by their parents. Immediately after cardiac surgery, drain tubes were inserted into pericardial space to relieve fluid accumulation around the heart. Drain fluids were collected every 24 h for 2 days after the operation in an aseptic container (Figure 1) containing 300 mL of cold saline and was kept at 4 °C. Cells were centrifuged at 1500 rpm for 10 min at 4 °C and re-suspended in 30 ml cold RPMI medium (Gibco, Invitrogen, Carlsbad, CA, USA). Mononuclear cells were isolated with Ficoll-Paque (GE Healthcare, Buckinghamshire, UK) by following the manufacturer’s instruction. They were cultured directly without sorting on a fibronectin-coated 48-well plate with cardiosphere medium [15] (300 µL/well) consisting of 35% IMDM and 65% DMEM/F-12 Mix (Gibco, Invitrogen, Carlsbad, CA, USA), 3.5% FBS, 1% penicillin-streptomycin, 1% L-glutamine, 0.1mM 2-mercaptoethanol, thrombin, 1× B-27, 80 ng/mL bFGF, 25 ng/mL EGF, and 4 ng/mL cardiotrophin-1. On the second and third day, 200 µL of the supernatant with RBCs and white blood cells were replaced carefully with freshly prepared medium without disturbing the loosely attached cells. The procedure was repeated every 3 days to completely remove the RBCs and unattached cells. Cells were expanded in a ratio of 1:2 in 5–7 days thereafter. 

### 2.2. Flow Cytometry

Cells isolated on day 1 and day 2 from cardiac surgery drain fluid were analyzed with flow cytometry to study the early surface marker expression. Cells with a higher passage (>5) were used to study the chronological changes of surface markers. All experiments were done with a FACSCalibur (BD Biosciences, San Jose, CA, USA) flow cytometer. Data were analyzed with free software WinMDI 2.8. Monoclonal antibodies used for cell surface markers analyses included fluorescein isothiocyanate (FITC)-conjugated anti-human CD34, phycoerythrin (PE)-conjugated anti-human CD133 (MACS, Bergisch Gladbach, Germany), allophycocyanin (APC)-conjugated anti-human CD117, PE-conjugated anti-human CD31, and CD45 (BD Pharmingen, San Diego, CA, USA), FITC-mouse IgG2aκ, APC-mouse IgG1κ, and PE-mouse IgG1κ isotype controls (BD Pharmingen, San Diego, CA, USA).

### 2.3. Cell Culture and Conditioned Medium for Angiogenesis Assay

Human fibroblasts from neonatal foreskin (HS68, Bioresource Collection and Research Center, Taipei, Taiwan) were cultured in DMEM with 10% fetal calf serum, 5% streptomycin and penicillin. Primary human umbilical vein endothelial cells (HUVEC) were purchased from the same center and were cultured in Medium 199 (Life Technologies, Taipei, Taiwan). Conditioned medium (CM) was collected 48 h in serum-free DMEM for fibroblasts, serum-free Medium 199 for HUVEC, and serum-free culture medium for PEPCs, respectively. CM was pooling from 100 µL of medium with 2 × 10^4^ cells in each well of a 96-well plate and stored at −20 °C until used. Twenty thousand HUVEC cells were seeded on a 96-well plate pre-coated with basement membrane matrix (Matrigel Matrix Growth Factor Reduced^TM^, BD Biosciences, Taipei, Taiwan). They were plated either with 100 µL complete Medium 199 as the positive control, with 100 µL FBS-free Medium 199 as the negative control, or with 100 µL CM. To evaluate the mechanism of angiogenesis elicited by PEPCs, neutralizing antibodies 0.04 μg/100 μL anti-VEGF (R&D, Minneapolis, MN, USA), or 0.8 μg/100 μL anti-HGF (Abcam, Billerica, MA, USA), or 50 µg/mL thalidomide (Sigma-Aldrich, St. Louis, MO, USA) was added to CM on a shaker for 1 hour at room temperature. Cells were imaged after 18 h with a digital camera coupled to a microscope. Five different areas were taken from one well. Images were analyzed with Image J software (Version 1.52p, National Institutes of Health, Bethesda, MD, USA) and the total vessel length was measured with AngioTool Software (version 0.6a, National Cancer Institute, Bethesda, MD, USA) [18]. Each condition was triplicated and at least three independent experiments were performed.

### 2.4. Cardiac Infarction in Mice

All animal studies were performed in compliance with the US Department of Health and Human Services Guide for the Care and Use of Laboratory Animals and approved by the institutional review board. Adult male SCID-beige mice, aged 10 to 20 weeks, were purchased from the animal center of National Taiwan University, Taipei, Taiwan. The left main descending branch of the coronary artery was ligated with a 7-0 prolene suture [15]. To minimize the number of mice used for the experiments and to compare results from different assays, PEPCs at passage 6 from three randomly selected patients were used in all following experiments. Either PEPCs or lentivirus-transduced PEPCs (10^5^ cells in a total volume of 10 µL phosphate-buffered saline (PBS)) were injected at two sites bordering the infarcts (5 µl each) immediately after vessels ligation. 8 mice were injected with PEPCs, while the other 6 mice were injected with lentivirus-transduced PEPCs for in vivo bioluminescence imaging. Transduced or non-transduced (both *n* = 8) human fetal skin fibroblasts (WS1, Bioresource Collection and Research Center, Taipei, Taiwan) and PBS (*n* = 11) were used as both positive and negative controls, respectively. WS1 is a normal skin fibroblast cell line originated from a 12-week gestation fetus. The embryonic nature of these cells is expected to have partial effects on tissue repair. These cells were grown in Eagle’s Minimum Essential Medium with 10% FBS.

All mice underwent echocardiography (Philips Sonos 5500, 15 MHz probe, Eindhoven, Netherlands) to evaluate the ventricular function on the day before operation (baseline, day 0), 2 days, 1 week, 2 weeks, and 3 weeks after surgery. Ventricular wall thickness and chamber dimensions were measured using a short-axis parasternal view at the level of the papillary muscles. Left ventricular ejection fraction (LVEF) and fractional area were calculated from 2D long-axis views taken through the infarcted area. Mice were sedated with isoflurane during all procedures. Data were collected by averaging three separate measurements during each examination. Mice were euthanized at 3 weeks.

### 2.5. Transplanted Cell Tracing with Optical Bioluminescence Imaging and Alu Sequence

Cardiac bioluminescence imaging was performed using the Xenogen IVIS 50 system (Hopkinton, MA, USA). After intraperitoneal injection of reporter probe D-luciferin (150 mg luciferin/kg), animals were imaged for 1–10 min under anesthesia. The same mice were scanned weekly for 3 weeks. Imaging signals were quantified in units of maximum photons per second per square centimeter per steradian (photons/sec/cm^2^/sr) as described [19]. To test the possibility that a small population of PEPCs might survive but not be detected by in vivo imaging, the human Alu repeat [20], acted as a marker to detect human PEPCs from infarcted hearts, was amplified by PCR.

### 2.6. Virus Production and Cell Transduction

PEPCs were transduced with luciferase gene as a tracer. The plasmid pLLB13 was constructed by inserting the Mef I-and Xba I-digested luciferase DNA fragment, PCR-amplified from pGL4.10 (Promega, USA) using both the 5’-primer (5′-CGAACTCAATTGCCACCATGGAAGATGCC-3′) and 3’primer (5′-CCCGACTCTAGAATTATTACACGGC-3′), into pLenti6/Ubc/V5-DEST (Invitrogen, Carlsbad, CA, USA) from which the 1752-bp fragment between EcoR I and Xba I restriction enzyme sites had been removed. Lentivirus production and transduction of PEPCs were performed as suggested by the manufacturer. Briefly, lentivirus was produced by transfecting 293FT cells (Invitrogen, Carlsbad, CA, USA) with both pLLB13 and ViroPower Packaging Mix (Invitrogen, Carlsbad, CA, USA) in the presence of Lipofectamine 2000 (Invitrogen, Carlsbad, CA, USA). The lentivirus was harvested three days later with a titer of 2 × 10^6^ transducing units. They were then used to transduce PEPCs in the presence of 6 µg/mL polybrene and 0.5 µg/mL blasticidin.

### 2.7. Immunocytochemistry and Histological Analysis

Spindle shape PEPCs and cardiosphere-like cell clusters were collected for immunostaining. Cells were fixed with 3.7% buffered paraformaldehyde, permeabilized with 0.5% Triton X-100 for 15 min and then blocked with goat serum (Zymed, San Diego, CA, USA) for 30 min at 37 °C. Cells were incubated with antibodies including mouse anti-CD105 (R&D Systems, Inc., Minneapolis, MN), anti-CX43 (Chemicon, Temecula, CA, USA), anti-sarcomeric actin (Abcam, Cambridge, UK), rabbit anti-CD117, anti-Ki67 (Abcam, Cambridge, UK) for 12 h at 4 °C, followed by an hour room temperature incubation with Alexa 488 or Alexa 489 conjugated secondary anti-mouse/anti-rabbit IgG antibodies (Molecular Probes, Eugene, OR, USA). Cell nucleus was stained with 4’, 6-diamidino-2-phenylindole (DAPI) (Sigma). The fluorophore was excited by a laser at 405 nm; 488 nm or 543 nm and detected by a scanning confocal microscope (Olympus FV-1000, Tokyo, Japan). The specificity of all antibodies used in this study was examined with isotype-specific immunoglobulins at the same protein concentration as a negative control. Mouse hearts were excised, fixed in 10% formaldehyde or fresh frozen, and sectioned in 5-μm slices. Tissue sections were stained with a hematoxylin-eosin reagent or Masson’s trichrome. Tissue infarct zone was calculated from Masson’s trichrome-stained sections by tracing the infarct borders manually [15] and then by using ImageJ software to calculate the percent of infarct myocardium by a researcher who blinded to the study.

### 2.8. Gene Expression in Mouse Heart after Cell Transplantation

To realize the crosstalk between implanted PEPCs and mouse cardiomyocytes, mouse hearts treated with PBS, human fibroblasts, and PEPCs were collected 3 weeks after surgery for DNA microarray analysis. Each group contained at least 3 mice. Mouse hearts were excised and frozen stored at −80 °C. The whole ventricular portion of each ischemic heart was used for RNA extraction. RNA was extracted after stabilizing in RNA later-ICE (Ambion, Foster City, CA, USA) by following the manufacturer’s instruction. Affymetrix cDNA microarray (Mouse Gene 1.0 ST Array GeneChip, Santa Clara, CA, USA) with 28,853 genes was used for whole-genome analysis. Gene expression level differences were analyzed with methods developed by Tusher VG et al. [21]. SYBR green master mix and 7500 Fast Real-Time PCR systems were used to confirm the gene expression (Applied Biosystems, Foster City, CA, USA). 18s RNA or GAPDH was used as an internal control.

### 2.9. Statistics Analysis

The Pearson product–moment correlation coefficient method was used to identify the patient parameter that might be independently associated with cell growth and cell isolation. One-way analysis of variance (ANOVA) was performed to determine whether there were significant differences between the different treatments. Bonferroni *t*-test was applied for multiple pairwise comparisons among individual groups. The Student’s *t*-test was used to compare differences in two groups when the variables were normally distributed and Mann–Whitney rank sum test when data were not normally distributed. Data were analyzed using SigmaStat (Systat Software Inc. San Jose, CA, USA) version 3.11. A *p*-value less than 0.05 was considered significant. Data were calculated with at least three separated independent experiments.

## 3. Results

### 3.1. Isolation of PEPCs from Drain Fluid after Open-Heart Surgery

**Figure 2:**
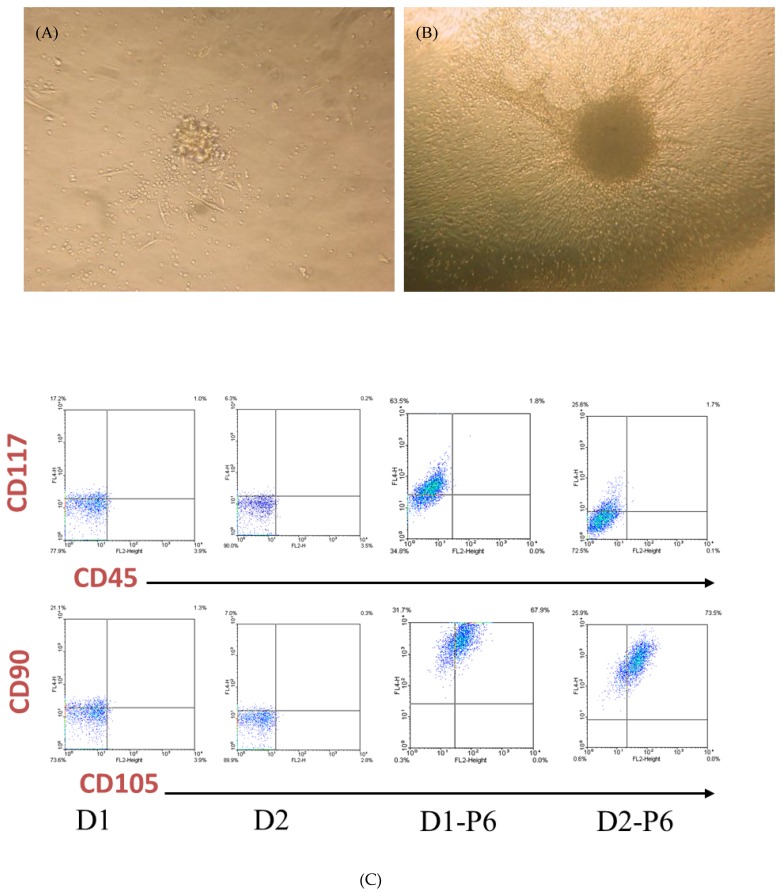

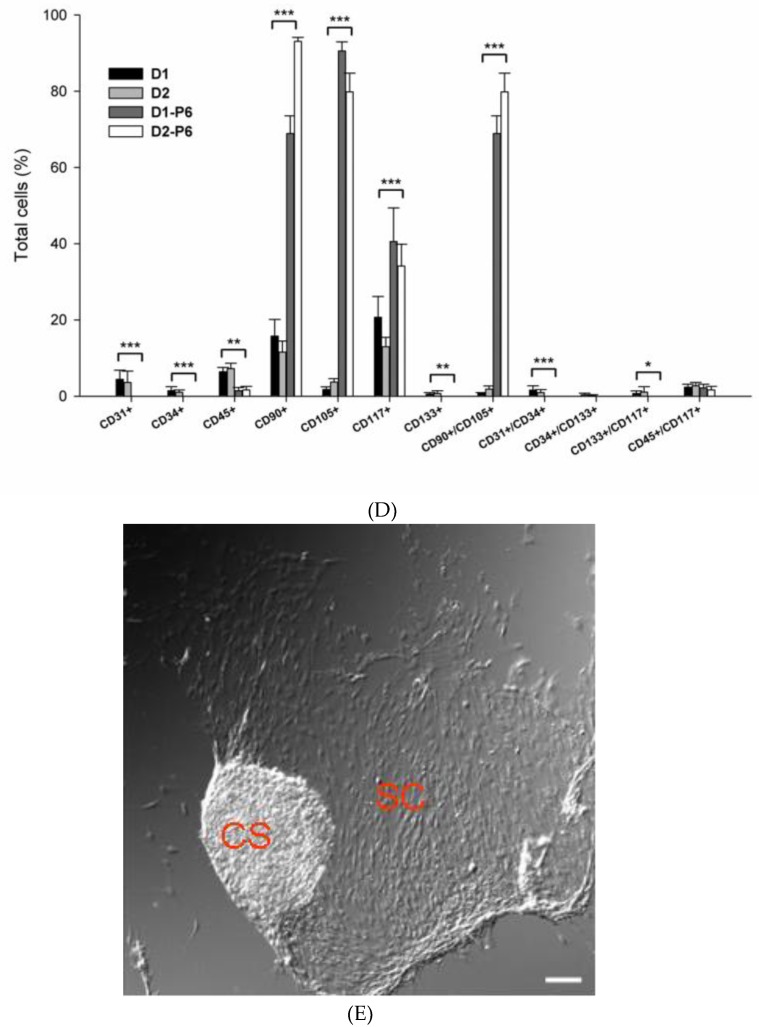

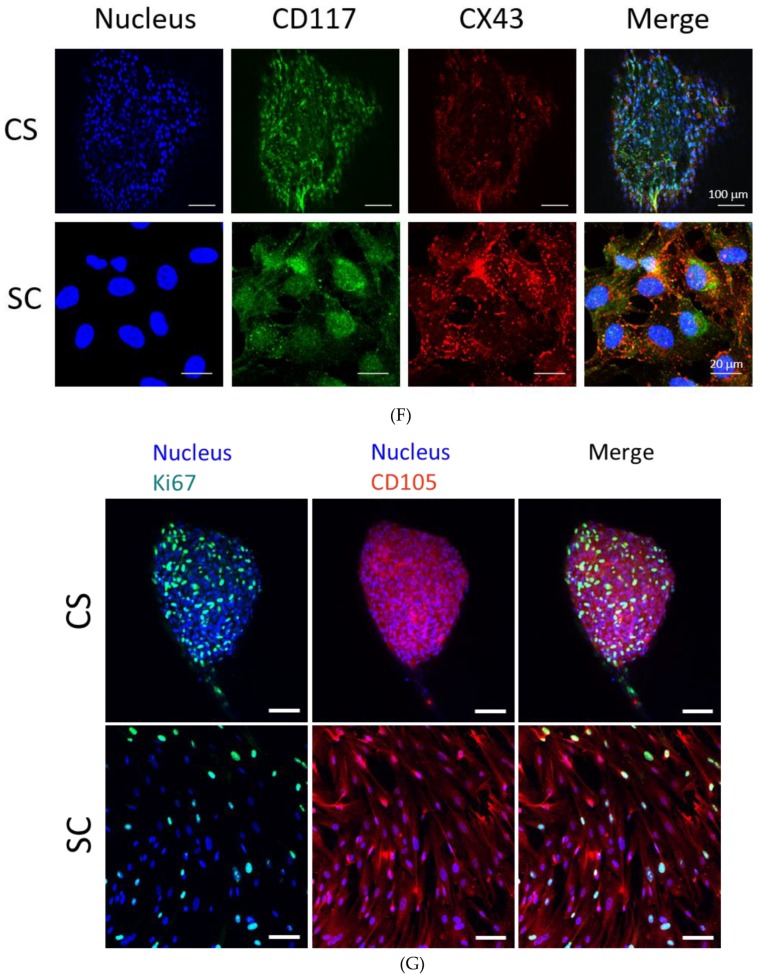

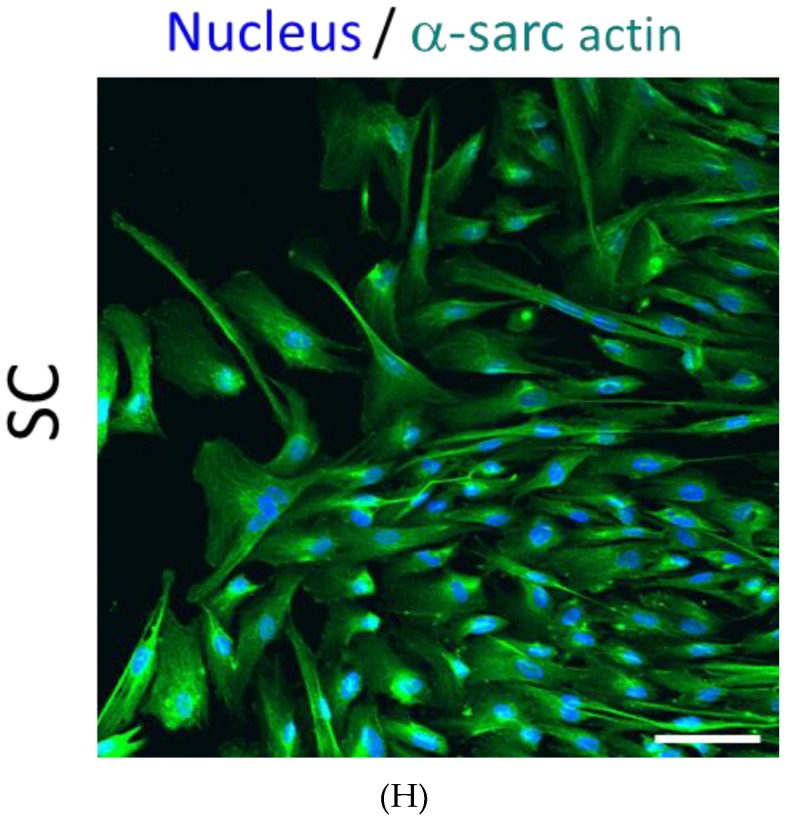
PEPCs are highly proliferating cells and express cardiac lineage potential after in vitro culture. (**A**) Spindle-like cells and cardiosphere-like cell clusters appeared 7 days after cell seeding. (**B**) Day 14 culture showed robust growth of spindle cells and cardiospheres. (**C**) FACS analysis, either from day 1 (D1) or day 2 (D2) drain fluid collections, showed increased expression of stem cell factor receptor (CD117), CD90, and CD105 after 6 cell passages (D1–P6, D2–P6) compared with their corresponding primary cells (D1 and D2). (**D**) The expression of CD31, CD34, CD45, and CD133 decreased with increased cell passages (* *p* < 0.05, ** *p* < 0.01, *** *p* < 0.001). (**E**) Differential interference contrast (DIC) image of cultured human PEPCs showed two distinct cell morphologies: cardiosphere-like (CS) and spindle-like cell (SC) on the fibronectin-coated plate. (**F**) and (**G**) Confocal immunofluorescence images of CS and SC cells stained with CD117, connexin (CX) 43, Ki67, CD105, (**H**) α-sarcomeric actin. Nuclei were counterstained with 4’, 6-diamidino-2-phenylindole (DAPI). Scale bar = 200 μm in E and G, and scale bar = 50μm in H.

Figure 1 depicts the methods of collection and propagation of PEPCs from the drain fluids. The average cells yield for the first 24-hour drain fluid was 6.3 ± 3.0 × 10^6^, and 2.2 ± 1.4 × 10^6^ for the second 24-hour. On 6–7 days-in-vitro (DIV), the morphology of the loosely attached cells changed. Spindle-like cells spread out on the culture dish while loosely attached cells began to form cell clumps resembling the morphology of a cardiosphere (Figure 2A). Subsequently, the culture showed robust growth. Spindle-like cells became confluent with cardiospheres growing on top on 14 DIV (Figure 2B). Of note, initial cell density affected cell growth [22]. With a higher culture density (2 × 10^4^/µL), spindle-like cells appeared on day 3 while cardiospheres appeared around day 7 after seeding. However, with a lower density (2 × 10^2^/µL), spindle-like cells appeared around day 7 while cardiopspheres emerged around 2–3 weeks. Importantly, PEPCs culture could be established from all 37 patients (3-day-old to 43-month-old, median 9-month-old) included in the study (Table S1), suggesting that this protocol was reproducible regardless of the patient’s sex, age, and disease conditions (r = 0, Pearson product–moment correlation coefficient). The doubling time of these cells was 4.56 ± 1.8 days.

### 3.2. PEPCs Differentiate toward Cardiovascular Lineage

PEPCs collected directly from patients’ day 1 or day 2 (*n* = 11 in each group) after surgery expressed similar, if not all, cell surface markers including CD31, CD34, CD45, CD90, CD105, CD117 (c-kit), and CD133 (Figure 2C,D) at variable levels (no significant difference, *p* > 0.05). Day 1 PEPCs contained surface markers including CD31 (4.4 ± 2.4%), CD34 (1.4 ± 1.2%), CD45 (6.4 ± 3.7%), CD90 (15.8 ± 12.3%), CD105 (1.8 ± 1.8%), CD117 (20.7 ± 18.1%), and CD133 (0.5 ± 0.4%), CD31/CD34 (1.6 ± 1.1%), CD34/CD133 (0.3 ± 0.4), CD45/CD117 (2.4 ± 2.5%), CD90/CD105 (0.7 ± 0.6%), CD117/CD133 (0.7 ± 0.7%). After six in vitro passages, the expressions of CD45 decreased to 1.4% while no expression of CD31, CD34, and CD133. CD90 (68.9%), CD105 (90.6%), and CD117 (40.6%) increased dramatically. Co-expression of surface markers CD45/CD117 was about the same (2.2 ± 1.0%) while CD90/CD105 increased significantly to 68.9 ± 4.7%. The present in vitro culture and passage condition might favor the expansion of a distinct population progenitor cells.

PEPCs including cardiospheres (CS) and spindle-like cells (SC) (Figure 2E) proliferated rapidly by a doubling time of 5–7 days during in vitro expansion. Consistent with this observation, immunofluorescence staining of Ki67 showed strong signals in both CS and SC (Figure 2G). PEPCs also expressed various cardiac stem cell and lineage-specific cell surface markers. In general, SC exhibited antigenic markers similar to those of the CS. As shown in Figure 2F, cells from both CS and SC populations express CD117, indicating the presence of progenitor cells [23]. The majority of PEPCs expressed markers indicative of cardiovascular lineages including CD105 [24], connexin (CX) 43 [25], and α-sarcomeric actin [15] (Figure 2F–H).

### 3.3. In Vitro Angiogenesis of PEPCs

**Figure 3:**
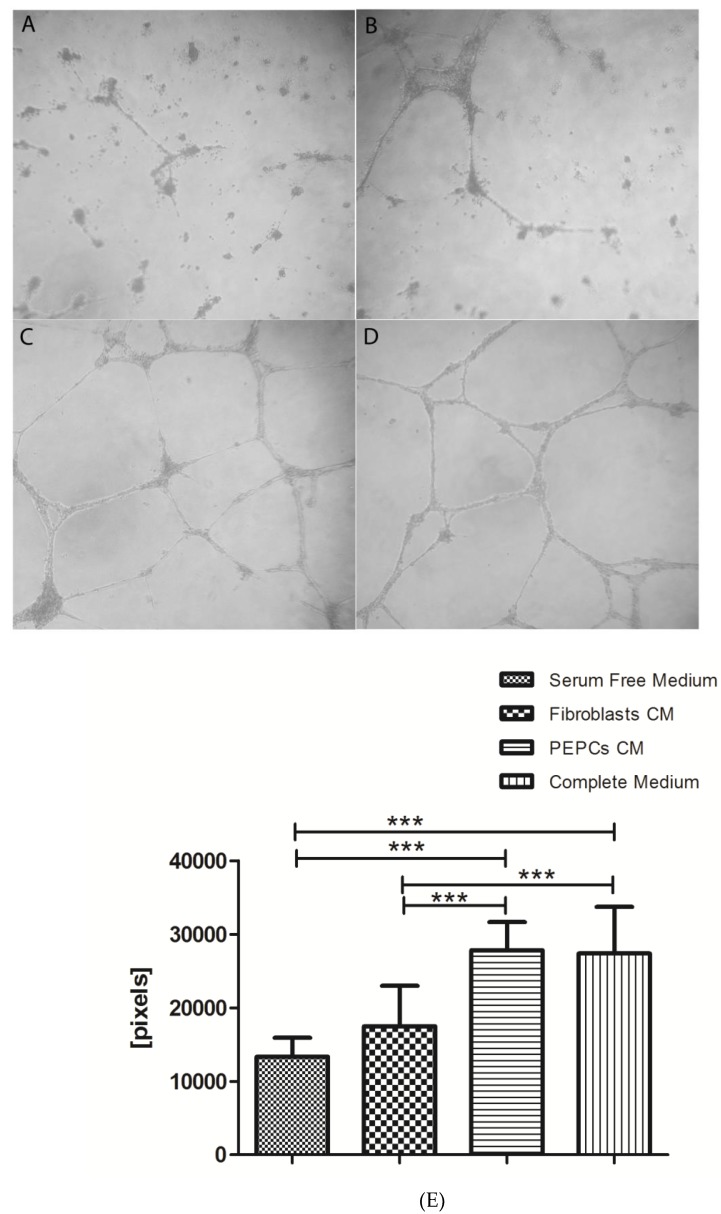

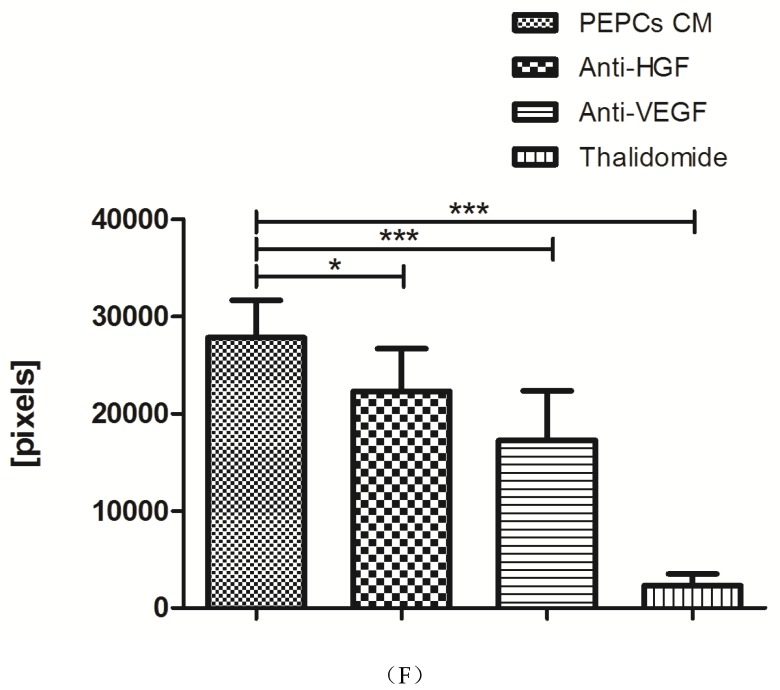
PEPCs conditioned medium enhanced angiogenesis in vitro. (**A**) Twenty thousand HUVEC cells were seeded on a 96-well plate pre-coated with basement membrane matrix. Cells were imaged after 18 h with a digital camera coupled to a microscope. HUVEC cells cultured in serum-free medium showed no tube formation. (**B**) Few vascular networks formed in serum-free conditioned medium from fibroblasts. (**C**) Well-formed vascular networks developed in cells cultured in PEPCs conditioned medium. (**D**) Cells cultured in completed medium (positive control) showed vascular networks as in PEPCs conditioned medium. (**E**) The total vessel length in pixels under different conditions. Data were pooled from three independent experiments expressed as average ± SD. (**F**) The enhancement was inhibited by anti-HGF, anti-VEGF and thalidomide (* *p* < 0.05; *** *p* < 0.001).

Conditioned medium from PEPCs enhanced angiogenesis of HUVEC in vitro. Figure 3 shows PEPCs condition medium significant enhanced HUVEC to form tubes (Figure 3C) in comparison to serum-free medium control (Figure 3A) or serum-free conditioned medium from fibroblasts (Figure 3B). The efficacy of PEPCs conditioned medium promoting tube formation was the same as medium supplemented with 10% serum (Figure 3D). The enhancement was inhibited by anti-HGF, anti-VEGF, and thalidomide (Figure 3E,F, * *p* < 0.05, *** *p* < 0.001).

### 3.4. Therapeutic Potential of PEPCs and Cell Fate in Ischemic Heart Repair

**Figure 4:**
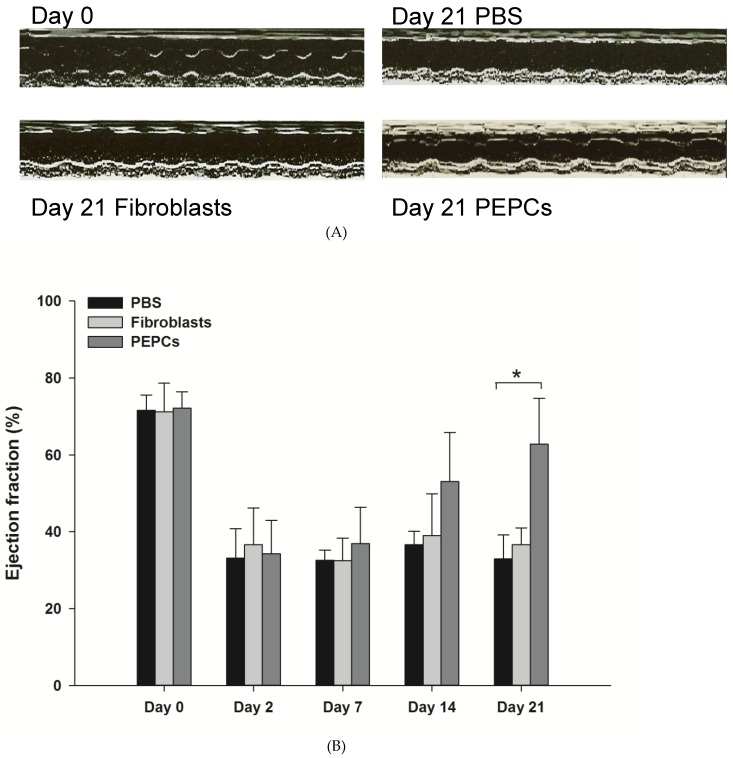

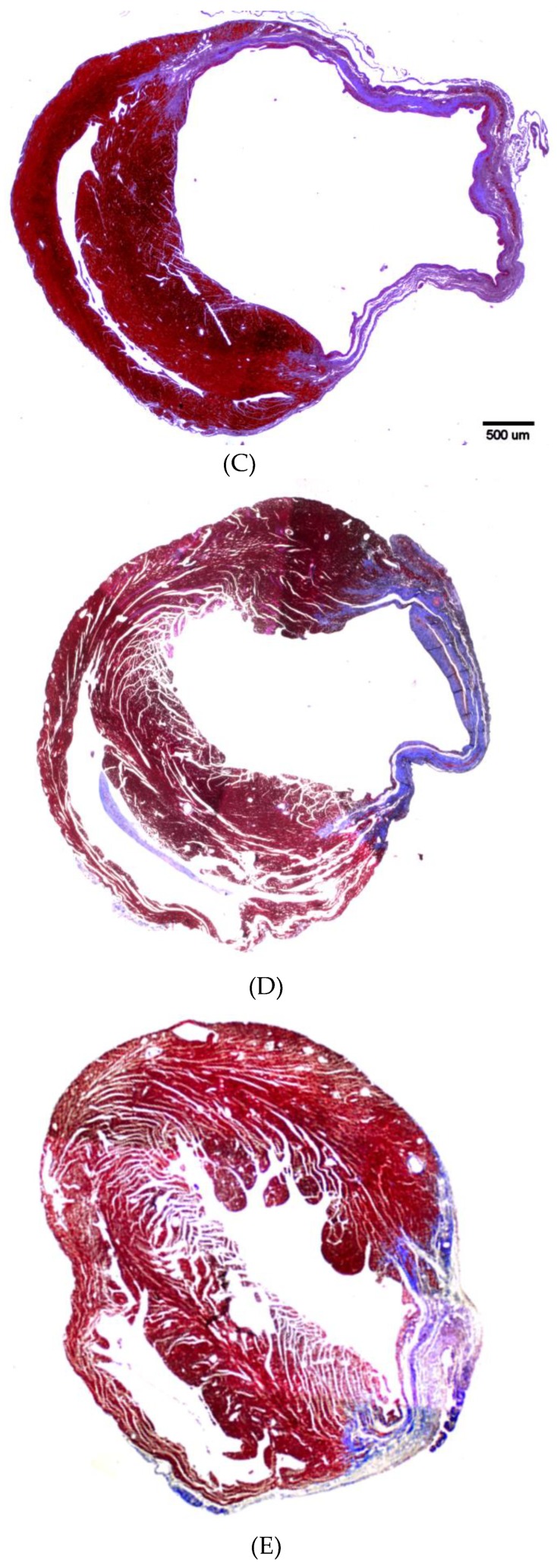

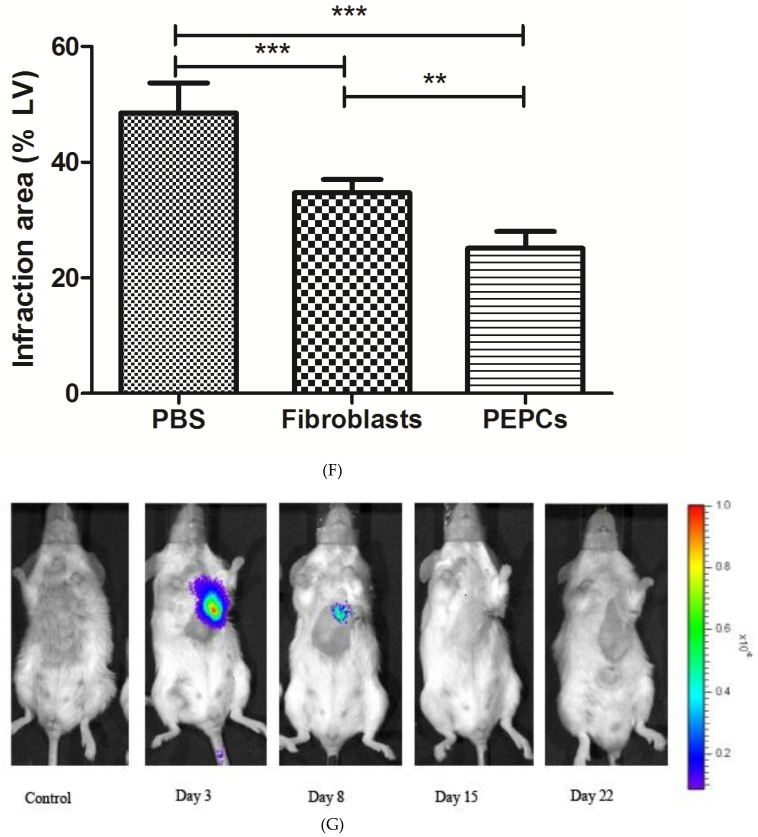

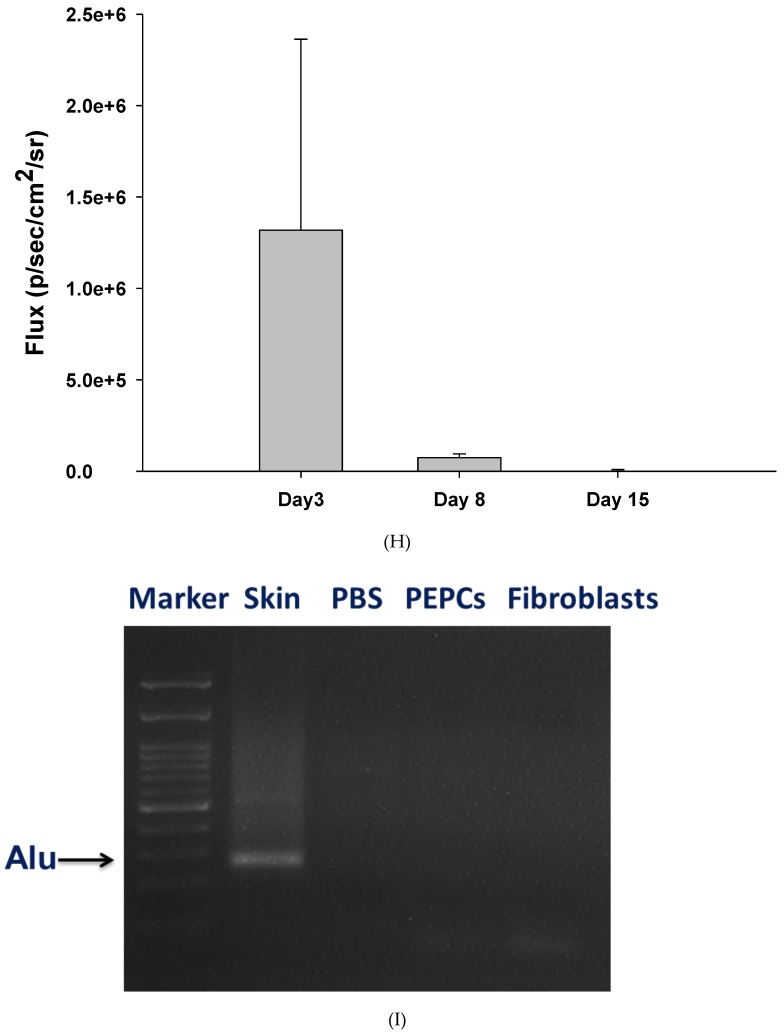
PEPCs restore cardiac functions and initiate tissue regeneration after transplantation into an ischemic heart. (**A**) Long-axis views from an echocardiogram performed 21 days post-infarction showed improvement of cardiac contractility after PEPCs transplantation (*n* = 14) compared to fibroblasts (*n* = 16) and PBS (*n* = 11). (**B**) Left ventricle ejection fractions were improved in mice injected with PEPCs (* *p* < 0.05). (**C**) Masson trichrome stain of ischemic heart in which viable tissue stains red whereas fibrous tissue stained blue, representative heart 3 weeks after PBS injection (*n* = 11); (**D**) fibroblasts transplanted (*n* = 10); and (**E**) PEPCs transplanted (*n* = 10). (**F**) Percent of infarction area in ischemic heart (** *p* < 0.01, *** *p* < 0.001) 3 weeks after cell transplantation. (**G**) In vivo imaging of PEPCs fate after transplantation. A representative mouse injected with 1 × 10^5^ PEPCs showed significant bioluminescence activity on day 3, which decreased progressively over the following 3 weeks. (**H**) Quantitative analysis of signals from all animals transplanted with PEPCs (signal activity is expressed as photons/sec/cm2/sr), *n* = 8 in PEPCs transplanted mice. (**I**) No human Alu sequence was detected in mouse heart 3 weeks after PEPCs transplantation.

Three weeks after injection of PEPCs into the margins of ischemic myocardium in mice, echocardiography revealed a significant improvement of cardiac ejection fraction compared to the PBS and fibroblast treated mice (Figure 4A,B; * *p* < 0.05). The regeneration ability was further quantified in sections stained with Masson trichrome to discriminate viable from fibrous tissue (Figure 4C–F). Ischemic hearts with PBS (negative control, Figure 4C) or fibroblasts (positive cell control, Figure 4D) transplantation showed significant heart dilatation. PEPC transplanted hearts had a smaller blue-stained (fibrous) infarct zone (25 ± 9%) compared to the hearts transplanted with fibroblasts (35 ± 7%; *p* < 0.01) or PBS-treated hearts (49 ± 17%; *** *p* < 0.001, Figure 4F).

In vivo luciferase signals from injected PEPCs declined from day 3 to day 15, and completely undetectable at day 22 after transplantation, advocating human PEPCs might not survive in injured mice hearts (Figure 4G,H). It may of interest to analyze whether any difference in cardiac repair ability between luciferase-transduced cells and non-transduced cells. However, the small sample size limited further subpopulation analysis. The absence of human cells was further confirmed by undetectable human Alu repeats in PEPC-treated infarct mouse heart (Figure 4I).

### 3.5. Paracrine Effects of PEPCs on Ischemic Heart Repair

**Figure 5:**
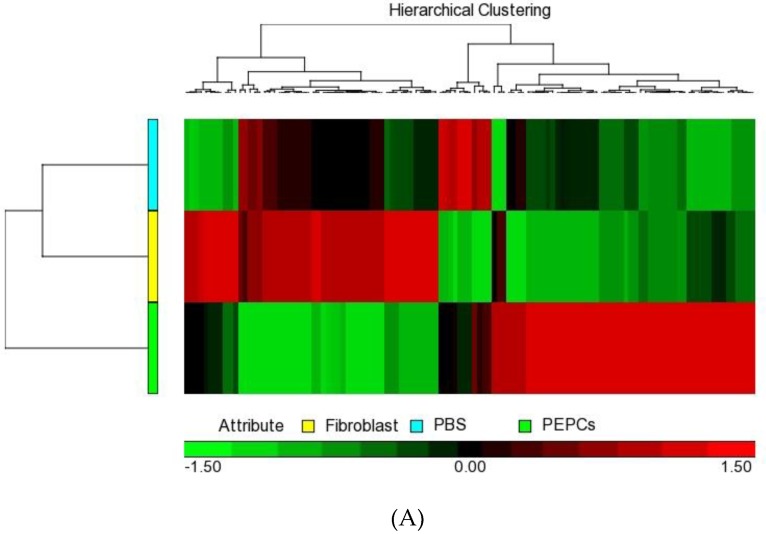

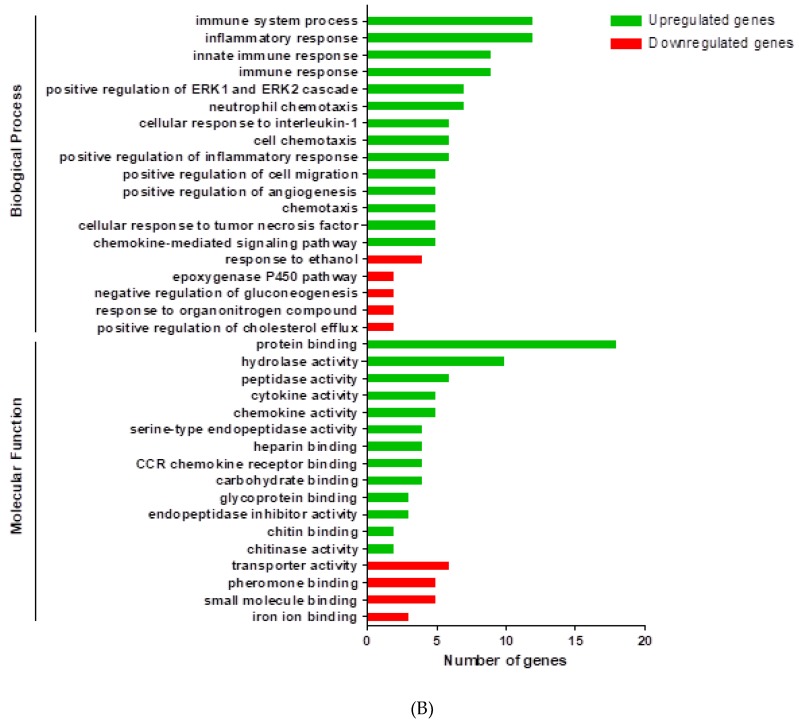
The difference of microarray gene expression profile after PEPCs-injected ischemic heart. (**A**) Hierarchical clustering heatmap displaying genes that were upregulated (red) or downregulated (green) in response to PEPCs, fibroblasts or PBS exposure (>1.5-fold change). (**B**) Gene ontology enrichment analysis of 95 differentially expressed genes revealed the enriched biological process and molecular function. Representative gene ontology terms listed on the bar plot have enrichment *p* values < 0.05 and the horizontal axis was the gene count.

To define the key components involved in the sustain effect of PEPCs on cardiac repair in the ischemic heart mouse model, we performed a whole genome microarray pooled from three ventricles of the mouse hearts in each group treated with PBS, human fibroblasts or PEPCs 22 days after transplantation. The heat map (baseline cutoff of ± 1.5fold change) indicated there are 95 genes regulated in the PEPCs-treated ischemic heart compared to human fibroblast-injected and PBS-injected controls (Figure 5A). Of these, 54 genes were upregulated and 41 genes were downregulated (Table S2).

Gene ontology analysis revealed several pathways to be significantly overrepresented in the PEPCs-treated group, including pathways involved in the immune and inflammatory response, cytokine and chemokine activity as well as paracrine signaling (Figure 5B). Particularly, genes involved in neutrophil chemotaxis such as chemokine ligands and cell adhesion such as integrins and cluster of differentiation molecules, were upregulated in PEPCs-treated ischemic hearts. Various cytokines including integrin subunit beta 2 (ITGB2) and matrix metalloproteinase-3 (MMP-3), which have been implicated in promoting angiogenesis and cell migration in the ischemic hearts [26] were increased in the PEPCs-treated group. The increased immune complements and chemokine receptors induced by PEPCs at a later time point when PEPCs were physically absent in the ischemic site suggested an enhanced homing or recruitment of host stem cells and other cell types that might contribute to ischemic heart repair [27].

## 4. Discussion

In this study, we found cells isolated from congenital heart disease patients’ own pericardial effusion for pressure relief after cardiac surgery may have potential to treat their heart disease in the future. We named these cells as PEPCs because the drainage tube was inserted in this space to relieve pressure after the operation. The PEPCs may be originated from heart, bone marrow or fibroblasts. Further studies are needed to clarify their origin. Agarwal U et al. found an age-dependent effect of human pediatric cardiac progenitor cells isolated from juvenile heart failure patients in repairing right ventricular heart failure in a rat [28]. They divided children undergoing reconstructive surgeries into three groups based on age: neonate (1 day to 1 month), infant (1 month to 1 year), and child (1 to 5 years). Injection of neonatal cardiac progenitor cells exerting the maximum beneficial effect compared with cells from infant and child. All patients in our study are younger than 4 years old (median 9 months). PEPCs could be established from all 37 patients regardless of age, sex, and disease types in our study. However, more patients are needed to confirm age effects in the future.

During the process of PEPCs culture, two critical points could determine the efficiency of PEPCs expansion: (1) initial cell density affects the cell growth and the formation of cardiospheres, and (2) a mixed cell population at the beginning of culture benefits cell growth. Initially, we tried to culture PEPCs after purifying the CD117^+^ cells through FACS cell sorting. Although CD117^+^ cells have been demonstrated to represent cardiac stem cells, this subpopulation of cells grew very slowly and failed to form cardiospheres. The unfavorable cell growth of this subpopulation might attribute to a low cell density and lack of cytokines/growth factors release from other cells. We decided to culture PEPCs without cell sorting. The suspended RBC and white blood cells were removed gradually by serial changing of the medium. A highly heterogeneous population of cells, possibly including inflammatory cells, mesenchymal stem/progenitor cells, and endothelial stem/progenitor cells at the initiation of PEPCs culture might provide a better milieu for subsequent PESCs expansion by mimicking host environment. This is in line with the observation by Smith RR et.al. in the establishment of cardiosphere-derived cells from cardiac biopsy without antigenic selection. The authors speculate that a mixed cell population might be advantageous for progenitor cell proliferation and function more potent than CD117 or CD90 purified stem cell subsets after injection into infarct areas [29]. It is also true in adipose tissue-derived stem cells in vitro [22]. Sukho P. et al. showed a higher cell seeding density in the presence of inflammatory cytokines enhances endothelial cell proliferation and fibroblast migration [22]. 

This study is focusing on how to isolate potential progenitor cells from surgery byproduct. The limitation of the present study is the lack of a sophisticated analysis of cell function and lineage assays by differentiation PEPCs with different culture media and a head-to-head comparison with another more established cardiac stem/progenitor cell. Nevertheless, PEPCs are heterogeneous in nature, exhibiting a phenotypic signature distinct from that reported for cardiac resident stem cell [15] and mesenchymal stem cells [16]. In the present study, day 1 and day 2 primary cells expressed CD117, the receptor for stem cell factor; and CD105, the regulatory component of the transforming growth factor-beta receptor complex which is important for angiogenesis [24]. A small portion (<5%) of cells expressed CD31. CD31 is also known as platelet/endothelial cell adhesion molecule-1 (PECAM-1) and can be found on the surface of platelets, monocytes, neutrophils, and some types of T-cells [30]. Less than 2% of cells expressed CD34 (hematopoietic progenitor cell antigen) which is associated with the selection and enrichment of hematopoietic stem cells for bone marrow transplants clinically but may express in different progenitor cells [31]. Around 7% PEPCs expressed CD45, a leukocyte common antigen and an essential regulator of T and B-cell antigen receptor signaling. The surface markers data implies a mixed cell population in the fresh isolates. Around 3% PEPCs expressed CD105 and 7% expressed CD45 while cardiac resident stem cells express almost 100% CD105 and do not express CD45 [15]. However, more than 70% PEPCs expressed CD90 and CD105 without expression of CD34 after in vitro expansion to passage 6. Our results are in agreement with recent studies that CD105^+^ MSC isolated from human heart improved ventricular ejection function in ischemic mouse heart and improved functional recovery by an ischemic limb after cells implantation [24]. MSC usually reveals a negative expression of CD34 and CD45 surface markers [32]. Colony-forming units-fibroblasts (CFU-Fs) also can repair myocardial damages [33]. The fibroblasts we used for in vivo study were originated from fetal skin. It was reasonable to expect some therapeutic effects in the ischemic heart as we showed in Figure 4D. However, these cells are CD117 negative cells. Taken together, we conclude that PEPCs may represent a distinct progenitor cell type. However, more studies are needed to clarify PEPCs as a new progenitor cell. 

The safety and efficacy of clinical trials of stem cell therapy in myocardial infarction patients are encouraging yet remain in dispute [7,16]. Despite the potential uses of ideally matched autologous cardiogenic cells from PEPCs transplantation, certain risks such as tumor formation and sudden death need to be concerned before clinical application. No tumors formed or sudden death happened in the PEPCs-treated mice. The use of cells with early passage (passage 6) for expansion may minimize the risk of tumor transformation of PEPCs [34,35]. However, a long-term follow up in vivo and more experiments are needed to confirm the results. 

The delay of cell harvesting to cell transplantation is one of the practical limitations in cell therapy. PEPCs expanded after six passages were used for cell therapy in eight ischemic hearts to achieve a homogenous cell background in mice. After expansion in vitro, 90% PEPCs expressed CD105 which may play a major role in therapeutic efficacy and cardiomyogenic differentiation [36]. The first 24 h drain fluid contained 6 million cells which may enough for cell therapy. It is of interest to study if the primary cells without expansion or cells with a lower passage expansion will have better or similar regenerative efficacy. We did not examine electrophysiology in PEPCs. The expression of connexin 43, a gap junction protein which plays a crucial role in the synchronized contraction, and α-sarcomeric actin, a major constituent of the contractile apparatus of cardiomyocytes [37,38] have been used as indicators for the electrical coupling potential [15]. PEPCs express both proteins, suggesting the potential to differentiate toward cardiomyocytes with electrically coupling potential. Nevertheless, long-term engraftment of implant cells may be unnecessary for cell therapy in cardiac repair and regeneration [29]. Indirect stimulation of the reparative response through paracrine effects has been one of the major hypotheses of cardiac regeneration in the first and second-generation cell therapy for myocardial infarction [16,39]. Bone marrow stem cell secretome analysis has revealed more than 100 soluble intrinsic paracrine factors, the restorative potential of which includes myocardial protection, angiogenesis, modulation of remodeling, increase in cardiomyocyte proliferation, and activation of resident progenitor cells [40]. The beneficial effects in reducing scars, reducing inflammation [41] and enhancing heart regeneration were mainly derived from indirect mechanisms such as paracrine effects [42], and exosomes secreted by cardiosphere-derived cells [43]. The recognition of dominant indirect mechanism opens a new research paradigm that secretory factors from stem cells may have potential in the regeneration of the ischemic heart [43]. However, the studying of the secretome of PECPs in vitro may not reflect the long-term effects in vivo, though it may explain the enhanced angiogenesis in Figure 3. The paracrine activity may require multiple cell type in the microenvironment. Therefore, we have used microarray to probe for differential gene expression in the infarction site. The microarray experiment at 3 weeks after transplantation revealed the upregulation of genes involved in chemotaxis, angiogenesis, and cell migration in PEPCs-transplanted heart, indicating that the long-term effect of PEPCs transplantation could trigger endogenous paracrine signaling that facilitates homing or recruitment of host stem cells and other cell types for ischemic heart repair.

## 5. Conclusions

In the present study, we have established a simple method for the isolation and propagation of progenitor cells from pericardial effusion which supposed to be discarded after heart surgery. Results from cardiac repair in mice suggested the potential of PEPCs in cell-based therapy in CHD. The PEPCs isolated and expanded by this method appear to have some pragmatic advantages. First, PEPCs are easy to harvest from drain fluids, the byproduct of open-heart surgery. Isolating and propagating cells without the need for cell sorting makes this technique simple and reduces the risk of contamination. Second, the yield of PEPCs is at least comparable, if not higher, to that previously reported for cardiosphere-derived cells obtained from endomyocardial biopsy (average 3 million in 4 weeks). Third, PEPCs are isolated from the same patient that will not raise an ethical issue and immune rejection for his/her future cell therapy. PEPCs may represent a new potential source for cell therapy in treating CHD with its easy harvesting protocol and potential in cardiac repairs. However, the functional characterization and differentiation of PEPCs into other cell types and the long-term safety needs to be investigated before the future application of PEPCs in the clinic.

## Supplementary Materials

The following are available online at https://www.mdpi.com/2077-0383/8/7/1028/s1, Table S1. Patients’ demographic data, Table S2. Differentially expressed genes in PEPCs-treated ischemic heart mice model.
