# Supplementary material for: Progenitor Cells Derived from Drain Waste Product of Open-Heart Surgery in Children
Source: J Clin Med. 2019 Jul 12;8(7):1028. doi: 10.3390/jcm8071028 (PMC6678880; doi:10.3390/jcm8071028)
Supplement: Supplementary file 1 [file jcm-08-01028-s001.pdf]

**Table S1.** Patients' demographic data.

| Patient | sex | Age | Disease | Cyanosis | Total bypass time (min) | Drain Volume (ml) |
|---|---|---|---|---|---|---|
| 1 | F | 11 M | Tetralogy of Fallot (TOF) | Y | 97 | 150 |
| 2 | M | 3 D | Transposition of great arteries (TGA) | Y | 95 | 65 |
| 3 | M | 9 M | Ventricular septal defect (VSD) | N | 49 | 45 |
| 4 | M | 20 M | VSD | N | 39 | 130 |
| 5 | M | 6 M | Pulmonary valve atresia with right ventricle hypoplasia | Y | 22 | 80 |
| 6 | F | 8 M | Partial anomalous pulmonary venous return with ASD | N | 36 | 50 |
| 7 | M | 7 D | TGA | Y | 104 | 85 |
| 8 | M | 4 M | VSD | N | 56 | 20 |
| 9 | F | 2 M | VSD | N | 67 | 47 |
| 10 | M | 7 M | Endocardial cushion defect | N | 93 | 80 |
| 11 | F | 1 M | Total anomalous pulmonary venous return | Y | 175 | 75 |
| 12 | M | 5 M | VSD | N | 52 | 20 |
| 13 | M | 15 M | TOF | Y | 103 | 20 |
| 14 | M | 9 M | Total anomalous pulmonary venous return | Y | 105 | 90 |
| 15 | M | 3 M | VSD | N | 44 | 40 |
| 16 | F | 34 M | TOF | Y | 96 | 35 |
| 17 | M | 26 M | VSD | N | 57 | 105 |
| 18 | M | 27 M | VSD | N | 64 | 90 |
| 19 | M | 8 D | Hypoplastic left heart syndrome | Y | 40 | 70 |
| 20 | M | 10 M | Total anomalous pulmonary venous return | Y | 43 | 78 |
| 21 | F | 10 M | TOF | Y | 107 | 130 |
| 22 | M | 3 M | VSD | N | 45 | 35 |
| 23 | M | 12 M | TOF | Y | 99 | 83 |
| 24 | M | 5 M | VSD | N | 66 | 18 |
| 25 | M | 32 M | Endocardial cushion defect | N | 70 | 50 |
| 26 | M | 3 M | VSD | N | 61 | 10 |
| 27 | M | 12 M | TOF | Y | 96 | 40 |
| 28 | M | 26 M | Endocardial cushion defect | N | 64 | 50 |
| 29 | M | 8 M | TOF | Y | 103 | 75 |
| 30 | F | 13 M | TOF | Y | 71 | 50 |
| 31 | M | 8 M | Right atrial isomerism | Y | 64 | 125 |
| 32 | M | 3 D | Hypoplastic left heart syndrome | Y | 50 | 100 |
| 33 | M | 5 D | TGA | Y | 148 | 20 |
| 34 | M | 2 M | VSD | N | 51 | 70 |
| 35 | M | 43 M | VSD | N | 44 | 50 |
| 36 | M | 19 M | TOF | Y | 89 | 200 |
| 37 | M | 2 M | VSD | N | 38 | 100 |

**Table S2.** Differentially expressed genes in PEPCs-treated ischemic heart mice model.

| Column number | Column ID | Gene Assignment | Gene Symbol | Fold-Change (PEPCs vs. Fibroblast) | Fold-Change (PEPCs vs. PBS) |
|---|---|---|---|---|---|
| 30086 | 10563597 | NM_011315//Saa3//serum amyloid A 3//7 B4\|7 30.51 cM//20210///ENSMUST000000069 | Saa3 | 3.62 | 5.86 |
| 25495 | 10521892 | NM_011402//Slc34a2//solute carrier family 34 (sodium phosphate), member 2//5 C1\|5 | Slc34a2 | 3.65 | 5.42 |
| 23266 | 10501020 | NM_009892//Chil3//chitinase-like 3//3 F2.2\|3 46.49 cM//12655///ENSMUST0000006 | Chil3 | 1.67 | 4.94 |
| 13976 | 10414202 | NM_023134//Sftpa1//surfactant associated protein A1//14 B\|14 22.36 cM//20387// | Sftpa1 | 1.60 | 4.87 |
| 7147 | 10349968 | NM_007695//Chil1//chitinase-like 1//1 E4\|1 58.15 cM//12654///XM_006529111// | Chil1 | 1.89 | 3.92 |
| 21198 | 10481962 | NM_010406//Hc//hemolytic complement//2 23.22 cM\|2 cen-C1//15139///ENSMUST0000 | Hc | 2.20 | 3.32 |
| 27253 | 10539091 | NM_001282071//Sftpb//surfactant associated protein B//6 C1\|6 32.27 cM//20388// | Sftpb | 2.58 | 2.51 |
| 22365 | 10492964 | NM_009690//Cd5l//CD5 antigen-like//3\|3 F1//11801///ENSMUST00000015998//Cd5l | Cd5l | 2.11 | 2.47 |
| 17862 | 10450325 | NM_001142706//Cfb//complement factor B//17 B1\|17 18.41 cM//14962///NM_008198 | Cfb | 2.76 | 2.35 |
| 7305 | 10351509 | NM_144559//Fcgr4//Fc receptor, IgG, low affinity IV//1 H3\|1 78.53 cM//246256// | Fcgr4 | 3.16 | 2.20 |
| 11211 | 10389222 | NM_009139//Ccl6//chemokine (C-C motif) ligand 6//11 C\|11 50.85 cM//20305///EN | Ccl6 | 1.72 | 2.14 |
| 15686 | 10429560 | NM_020498//Ly6i//lymphocyte antigen 6 complex, locus I//15\|15 D3//57248///XM_ | Ly6i | 1.88 | 2.13 |
| 11210 | 10389214 | NM_011338//Ccl9//chemokine (C-C motif) ligand 9//11 C\|11 50.81 cM//20308///EN | Ccl9 | 1.63 | 2.05 |
| 17222 | 10444407 | NM_001271422//Ager//advanced glycosylation end product-specific receptor//17\|17 B | Ager | 1.64 | 2.04 |
| 10217 | 10379535 | NM_021443//Ccl8//chemokine (C-C motif) ligand 8//11 C\|11 49.91 cM//20307///AB | Ccl8 | 1.59 | 1.97 |
| 33803 | 10596014 | NM_001194921//Cldn18//claudin 18//9\|9 E3-F1//56492///NM_001194922//Cldn18/ | Cldn18 | 1.56 | 1.97 |
| 28220 | 10547664 | NM_019948//Clec4e//C-type lectin domain family 4, member e//6 58.35 cM\|6 F3//56 | Clec4e | 1.93 | 1.93 |
| 26444 | 10531415 | NM_021274//Cxcl10//chemokine (C-X-C motif) ligand 10//5 E2\|5 46.57 cM//15945// | Cxcl10 | 4.01 | 1.90 |
| 32363 | 10582580 | NR_046144//n-R5s136//nuclear encoded rRNA 5S 136//8 E\|8 72.1 cM//100861491 | n-R5s136 | 1.52 | 1.89 |
| 33853 | 10596403 | NM_001167923//Col6a5//collagen, type VI, alpha 5//9 F1\|9//665033///ENSMUST000 | Col6a5 | 2.58 | 1.88 |
| 8648 | 10364262 | NM_008404//Itgb2//integrin beta 2//10 C1\|10 39.72 cM//16414///XM_006513271// | Itgb2 | 3.03 | 1.85 |
| 22408 | 10493474 | NM_013605//Muc1//mucin 1, transmembrane//3 F1\|3 39.02 cM//17829///ENSMUST0000 | Muc1 | 1.62 | 1.84 |
| 12065 | 10398075 | NM_009252//Serpina3n//serine (or cysteine) peptidase inhibitor, clade A, member 3N | Serpina3n | 2.26 | 1.81 |
| 18088 | 10452316 | NM_009778//C3//complement component 3//17 29.72 cM\|17 E1-E3//12266///XM_01124 | C3 | 3.56 | 1.81 |
| 8190 | 10360070 | NM_010185//Fcer1g//Fc receptor, IgE, high affinity I, gamma polypeptide//1 H3\|1 7 | Fcer1g | 1.97 | 1.79 |

| | | | | | |
|---|---|---|---|---|---|
| 26777 | 10534927 | NM_153510//Pilra//paired immunoglobin-like type 2 receptor alpha//5|5 G2//23180 | Pilra | 1.76 | 1.76 |
| 21907 | 10488804 | ENSMUST00000184654//Gm14214//predicted gene 14214 [Source:MGI Symbol;Acc:MGI:364935 | Gm14214 | 1.70 | 1.74 |
| 32023 | 10579347 | NM_023065//Ifi30//interferon gamma inducible protein 30//8|8 B3.3//65972///EN | Ifi30 | 1.98 | 1.71 |
| 7303 | 10351504 | XM_011238892//Gm39701//predicted gene, 39701//1|1//105244006 | Gm39701 | 1.66 | 1.66 |
| 29445 | 10557862 | NM_001082960//Itgam//integrin alpha M//7|7 F4//16409///NM_008401//Itgam// | Itgam | 1.82 | 1.66 |
| 25469 | 10521667 | NM_009763//Bst1//bone marrow stromal cell antigen 1//5 B3|5 23.84 cM//12182/// | Bst1 | 1.53 | 1.66 |
| 26060 | 10527638 | NM_001308462//Alox5ap//arachidonate 5-lipoxygenase activating protein//5|5 G3// | Alox5ap | 1.64 | 1.64 |
| 34740 | 10603551 | NM_007807//Cybb//cytochrome b-245, beta polypeptide//X|X A1.1//13058///XM_006 | Cybb | 2.47 | 1.64 |
| 29086 | 10554789 | NM_001311790//Ctsc//cathepsin C//7|7 D3-E1.1//13032///NM_009982//Ctsc//ca | Ctsc | 1.68 | 1.63 |
| 8179 | 10359999 | ENSMUST00000180190//Gm23523//predicted gene, 23523 [Source:MGI Symbol;Acc:MGI:54533 | Gm23523 | 1.63 | 1.63 |
| 30811 | 10568024 | NM_001301374//Coro1a//coronin, actin binding protein 1A//7 69.25 cM|7 F3//12721 | Coro1a | 1.85 | 1.62 |
| 12575 | 10402406 | NM_009245//Serpina1c//serine (or cysteine) peptidase inhibitor, clade A, member 1C | Serpina1c | 2.19 | 1.62 |
| 27126 | 10538150 | NM_001098271//Tmem176a//transmembrane protein 176A//6|6 B2.3//66058///NM_0253 | Tmem176a | 1.51 | 1.62 |
| 11213 | 10389231 | NM_011337//Ccl3//chemokine (C-C motif) ligand 3//11 C|11 51.04 cM//20302///EN | Ccl3 | 2.39 | 1.62 |
| 25033 | 10517165 | NM_013706//Cd52//CD52 antigen//4 D3|4 66.5 cM//23833///ENSMUST00000000696// | Cd52 | 1.85 | 1.61 |
| 12187 | 10398907 | NM_178911//Pld4//phospholipase D family, member 4//12|12 F1//104759///XM_0112 | Pld4 | 2.29 | 1.60 |
| 22513 | 10494271 | NM_001267695//Ctss//cathepsin S//3 F2.1|3 40.74 cM//13040///NM_021281//Ctss | Ctss | 1.98 | 1.59 |
| 26588 | 10532744 | NM_009151//Selplg//selectin, platelet (p-selectin) ligand//5 F|5 55.59 cM//2034 | Selplg | 1.91 | 1.58 |
| 32426 | 10583071 | NM_010809//Mmp3//matrix metallopeptidase 3//9 A1|9 2.46 cM//17392///ENSMUST00 | Mmp3 | 1.52 | 1.58 |
| 29593 | 10559467 | XM_003084682//Gm15448//predicted gene 15448//7|7 A1//100041146///XM_006540448 | Gm15448 | 2.09 | 1.56 |
| 12262 | 10399581 | XR_868554//3110053B16Rik//RIKEN cDNA 3110053B16 gene//12|12 A1.2//382686///XR | 3110053B16 Rik | 1.54 | 1.54 |
| 27523 | 10541683 | NM_001113356//C1rb//complement component 1, r subcomponent B//6 F2|6//667277// | C1rb | 2.97 | 1.54 |
| 7296 | 10351477 | NM_012009//Sh2d1b1//SH2 domain containing 1B1//1|1 H2//26904///ENSMUST0000017 | Sh2d1b1 | 1.99 | 1.54 |
| 18459 | 10456005 | NM_001042605//Cd74//CD74 antigen (invariant polypeptide of major histocompatibility | Cd74 | 2.91 | 1.54 |
| 9097 | 10368343 | NM_007482//Arg1//arginase, liver//10|10 A4//11846///ENSMUST00000020161//Arg | Arg1 | 1.64 | 1.53 |

| | | | | | |
|---|---|---|---|---|---|
| 32239 | 10581434 | NM_001301204//Dpep2//dipeptidase 2//8\|8 D3//319446///NM_001301205//Dpep2// | Dpep2 | 2.12 | 1.53 |
| 17214 | 10444298 | NM_010382//H2-Eb1//histocompatibility 2, class II antigen E beta//17 B1\|17 17.98 | H2-Eb1 | 2.12 | 1.52 |
| 28657 | 10551025 | NM_007655//Cd79a//CD79A antigen (immunoglobulin-associated alpha)//7 A3\|7 13.49 c | Cd79a | 1.64 | 1.51 |
| 25067 | 10517508 | NM_009777//C1qb//complement component 1, q subcomponent, beta polypeptide//4 D3\|4 | C1qb | 1.82 | 1.50 |
| 35196 | 10606868 | NM_009052//Bex1//brain expressed gene 1//X F1\|X 57.4 cM//19716///ENSMUST00000 | Bex1 | -1.52 | -1.50 |
| 22128 | 10490838 | NM_001272097//Fabp5//fatty acid binding protein 5, epidermal//3\|3 A1-A3//16592 | Fabp5 | -2.06 | -1.50 |
| 16593 | 10438681 | NM_001102409//Kng2//kininogen 2//16\|16 B1//385643///NM_001102410//Kng2//k | Kng2 | -3.35 | -1.51 |
| 14574 | 10419082 | NM_001316732//Fam213a//family with sequence similarity 213, member A//14\|14 B// | Fam213a | -2.22 | -1.52 |
| 28686 | 10551282 | NM_007812//Cyp2a5//cytochrome P450, family 2, subfamily a, polypeptide 5//7 A3\|7 | Cyp2a5 | -8.19 | -1.52 |
| 8442 | 10362404 | NM_029726//Trdn//triadin//10\|10 A4//76757///ENSMUST00000095762//Trdn//tri | Trdn | -2.20 | -1.53 |
| 17353 | 10445428 | ENSMUST00000083809//Gm25008//predicted gene, 25008 [Source:MGI Symbol;Acc:MGI:54547 | Gm25008 | -4.69 | -1.53 |
| 34026 | 10598081 | ENSMUST00000082401//mt-Ty//mitochondrially encoded tRNA tyrosine [Source:MGI Symbol | mt-Ty | -1.76 | -1.54 |
| 6858 | 10346914 | NM_172422//Fastkd2//FAST kinase domains 2//1\|1 C2//75619///XM_006496313//Fa | Fastkd2 | -2.00 | -1.54 |
| 30458 | 10565609 | NM_009381//Thrsp//thyroid hormone responsive//7\|7 E3//21835///ENSMUST00000043 | Thrsp | -5.96 | -1.54 |
| 16513 | 10437942 | NM_001159351//Ube2v2//ubiquitin-conjugating enzyme E2 variant 2//16\|16 A1//7062 | Ube2v2 | -2.17 | -1.55 |
| 8455 | 10362446 | NM_029726//Trdn//triadin//10\|10 A4//76757///ENSMUST00000095762//Trdn//tri | Trdn | -2.26 | -1.58 |
| 8447 | 10362424 | NM_029726//Trdn//triadin//10\|10 A4//76757///ENSMUST00000095762//Trdn//tri | Trdn | -2.39 | -1.59 |
| 8446 | 10362422 | NM_029726//Trdn//triadin//10\|10 A4//76757///ENSMUST00000095762//Trdn//tri | Trdn | -2.03 | -1.60 |
| 18750 | 10458828 | NM_033037//Cdo1//cysteine dioxygenase 1, cytosolic//18 C\|18 24.75 cM//12583/// | Cdo1 | -9.66 | -1.60 |
| 8458 | 10362454 | NM_029726//Trdn//triadin//10\|10 A4//76757///ENSMUST00000095762//Trdn//tri | Trdn | -3.73 | -1.60 |
| 34025 | 10598079 | ENSMUST00000082400//mt-Tc//mitochondrially encoded tRNA cysteine [Source:MGI Symbol | mt-Tc | -2.18 | -1.62 |
| 8506 | 10362896 | NM_009846//Cd24a//CD24a antigen//10 B2\|10 23.01 cM//12484///ENSMUST0000005871 | Cd24a | -2.58 | -1.63 |
| 23780 | 10505438 | NM_008768//Orm1//orosomucoid 1//4 B3\|4 33.96 cM//18405///ENSMUST00000030044/ | Orm1 | -13.17 | -1.64 |
| 24614 | 10513504 | NM_001199936//Mup16//major urinary protein 16//4 B3\|4//100039177///XM_0065374 | Mup16 | -2.56 | -1.64 |
| 8444 | 10362418 | NM_029726//Trdn//triadin//10\|10 A4//76757///ENSMUST00000095762//Trdn//tri | Trdn | -2.26 | -1.66 |
| 27682 | 10542983 | NM_011134//Pon1//paraoxonase 1//6 1.99 cM\|6 A2//18979///XM_006505011//Pon1 | Pon1 | -3.94 | -1.67 |
| 24611 | 10513467 | NM_001134674//Mup13//major urinary protein 13//4 B3\|4//100039089///XM_0065374 | Mup13 | -3.11 | -1.79 |

| | | | | | |
|---|---|---|---|---|---|
| 24609 | 10513437 | NM_001122647//Mup10//major urinary protein 10//4\|4 B3//100039008///NM_0011346 | Mup10 | -3.20 | -1.81 |
| 24607 | 10513420 | NM_001134675//Mup7//major urinary protein 7//4 B3\|4//100041658///XM_006536396 | Mup7 | -3.58 | -1.85 |
| 28155 | 10546929 | NM_001301295//Cidec//cell death-inducing DFFA-like effector c//6\|6 E3//14311// | Cidec | -3.19 | -1.86 |
| 24610 | 10513455 | NM_001134676//Mup8//major urinary protein 8//4 B3\|4//100041687///NM_001163010 | Mup8 | -3.58 | -1.90 |
| 29341 | 10556769 | NM_016870//Acsm3//acyl-CoA synthetase medium-chain family member 3//7\|7 F3//202 | Acsm3 | -7.05 | -1.95 |
| 31019 | 10569870 | NM_001204959//Retn//resistin//8 1.92 cM\|8 A1//57264///NM_022984//Retn//re | Retn | -5.20 | -1.97 |
| 8457 | 10362450 | NM_029726//Trdn//triadin//10\|10 A4//76757///ENSMUST00000095762//Trdn//tri | Trdn | -3.09 | -2.02 |
| 9512 | 10372730 | NM_054079//Iltifb//interleukin 10-related T cell-derived inducible factor beta//1 | Iltifb | -1.75 | -2.04 |
| 34027 | 10598083 | ENSMUST00000082403//mt-Ts1//mitochondrially encoded tRNA serine 1 [Source:MGI Symbo | mt-Ts1 | -3.03 | -2.05 |
| 8696 | 10364542 | NM_001291915//Cfd//complement factor D (adipsin)//10 C1\|10 39.72 cM//11537/// | Cfd | -4.21 | -2.14 |
| 33387 | 10592044 | NM_144936//Tmem45b//transmembrane protein 45b//9\|9 A4//235135///XM_011242482 | Tmem45b | -19.14 | -2.19 |
| 7692 | 10355259 | NM_001113387//Myl1//myosin, light polypeptide 1//1 C3\|1 33.71 cM//17901///NM_ | Myl1 | -1.98 | -2.21 |
| 27956 | 10545210 | K00746//Igkv4-55//immunoglobulin kappa variable 4-55//6\|6 C1//385253///OTTMUS | Igkv4-55 | -2.26 | -2.26 |
| 34029 | 10598087 | ENSMUST00000082419//ND6//NADH dehydrogenase subunit 6//---//17722 | ND6 | -3.22 | -2.35 |
| 16173 | 10434747 | NM_009605//Adipoq//adiponectin, C1Q and collagen domain containing//16 B3-B4\|16 1 | Adipoq | -11.39 | -3.08 |
| 8453 | 10362440 | NM_029726//Trdn//triadin//10\|10 A4//76757///ENSMUST00000095762//Trdn//tri | Trdn | -7.02 | -3.86 |
| 29521 | 10558673 | NM_021282//Cyp2e1//cytochrome P450, family 2, subfamily e, polypeptide 1//7 F5\|7 | Cyp2e1 | -8.03 | -6.22 |
| 22138 | 10490913 | NM_007606//Car3//carbonic anhydrase 3//3 3.22 cM\|3 A2//12350///XM_011248138/ | Car3 | -26.05 | -8.49 |
